# Life-LCA: case study of the life cycle impacts of an infant

**DOI:** 10.1007/s11367-022-02129-7

**Published:** 2023-01-14

**Authors:** David Bossek, Vanessa Bach, Matthias Finkbeiner

**Affiliations:** Chair of Sustainable Engineering, Technische Universität Berlin, Strasse des 17. Juni 135, 10623 Berlin, Germany

**Keywords:** Life cycle assessment (LCA), Life-LCA, Sustainable consumption, Infant, Environmental footprint, Carbon footprint, Prenatal, Sustainable lifestyles, Environmental awareness

## Abstract

**Purpose:**

The recently published first Life-LCA case study of a human being (0–49 years) did not use primary data for the “childhood and youth stage” (0–17 years). Consumption was assumed to contribute 50% of the calculated 48^th^ baseline year. This led to uncertainties as consumer behavior changes from birth to adulthood. Furthermore, transport emissions and environmental impacts before birth were neglected. Therefore, this paper analyzes the prenatal and infancy phase (0–3 years) to develop the Life-LCA method and database further and evaluate generic assumptions.

**Methods:**

The Life-LCA method sets the reporting unit to newly defined prenatal and infancy phases. The reporting flow describes the range of all consumed products attributable to an infant. Primary data was collected with a sample of three study objects—a pregnant mother, a newborn baby, and a 3-year-old infant—living in Germany. The following environmental impact assessment categories are considered: climate change (GWP), acidification (AP), eutrophication (EP), and photochemical ozone creation (POCP).

**Results and discussion:**

Prenatal and infancy phase burdens account for a GWP of 4,011 kg CO_2_-eq., an AP of 22.3 kg SO_2_-eq., an EP of 10.7 kg PO_4_-eq., and a POCP of 1.7 kg C_2_H_4_-eq. The share of the prenatal phase is around 15–20% for all impact categories. Transport is a hotspot for GWP (30–60%) and POCP (45–70%) in both phases. AP (50%) and EP (45–50%) are dominated by food products, mainly meat (45%) and dairy products (35%). For the prenatal phase, energy and water consumption at birth rank third in GWP (8%). Diapers account for 6% (GWP) of the environmental burden in the infancy phase. Assumptions made in the first Life-LCA study connect closely with the values calculated for the first three years of infancy. A remaining challenge is allocating the impacts between infants and parents and developing a methodology for assessing data quality.

**Conclusion:**

Focusing on two new life phases has led to the subdivision of the “childhood and youth stage” and an extension of the system boundaries. The results' uncertainty was reduced by developing a new set of specific datasets focusing on several study objects. The case study results show the importance of primary data collection for evaluating generic assumptions. Additional studies on childhood and adolescence from 3 to 17 years are suggested for a robust assessment of the complete “childhood and youth stage.”

**Supplementary Information:**

The online version contains supplementary material available at 10.1007/s11367-022-02129-7.

## Introduction

Due to advanced medicine and the industrial revolution, the human population gains around 385,000 lives daily. It is estimated that by 2030, humanity will number around 8.5 billion human beings living on earth (United Nations 2015). This fundamental human development puts enormous pressure on the planet and its available resources (Galvani et al. 2016). Therefore, consumer responsibility and early implemented sustainable consumption patterns are essential, as described in Sustainable Development Goal 12 (United Nations 2016).

In the first Life-LCA case study (Bossek et al. 2021) of a human being (referred to as the “first Life-LCA case study” in the remainder of this paper), the environmental impacts caused by a German male from birth until his current age (0–49 years) were determined by applying the Life-LCA method (Goermer et al. 2020), including possible reduction potentials. However, it was not possible to collect primary data for all life cycle stages. Thus, it was assumed that 50% of the environmental impacts of the baseline year (48^th^ life year) for every product category (excluding transport) correspond with consumption per year in the “childhood and youth stage” (birth to 17 years). This retrospective approach led to uncertainties in the overall results as consumer behavior changes from birth to adulthood, e.g., food intake or clothes (ANSES 2017), and infants depend on their parents’ consumer choices. Due to the absence of case studies, the environmental impacts during the “childhood and youth stage” and especially years as an infant have not been quantified so far. Therefore, this case study carries out a necessary primary data collection to be able to make statements about the potential environmental impacts of this life stage and evaluate the made assumptions, considering a fast-changing and complex consumer behavior for growing infants. Instead of covering the entire “childhood and youth stage,” the results’ accuracy is enhanced by dividing the life stage into several life phases. Numerous publications classify human development phases from birth to adulthood (e.g., Erikson 1963; Bogin 2015; Balasundaram and Avulakunta 2021). Besides Moore et al. (2013), the reviewed studies do not start their classification before birth or consider an infant’s prenatal burdens. However, this case study extends the “childhood and youth stage” defined by Goermer et al. (2020) from birth to 17 years to a new “prenatal phase” to begin emission accounting at the very beginning of a human being’s life. Furthermore, Bogin (2015) classified a “neonatal period” of 28 days, “infancy” as up to 3 years, and “childhood” from ages three to seven. The author further describes “juvenility years” from ages 7–12, followed by an adolescence phase lasting from 5 to 10 years. In contrast, Erikson (1963), who specialized in psychosocial development, separated the infancy phase as defined by Bogin (2015) into “infancy” (ages 0–1 ½) and “early childhood” (ages 1 ½ –3), followed by play age (ages 3–5), school-age (ages 5–12), and adolescence (ages 12–18). However, Balasundaram and Avulakunta (2021) categorize into infancy (ages 0–1), toddler (ages 1–5), childhood (ages 3–11), and adolescence phase from ages 12–18. As all studies agree on the ages 12–18 as “adolescence,” this classification is used for Life-LCA. Furthermore, considering the above mentioned, the “infancy phase” is defined as from birth to 3 years based on Moore et al. (2013) and Bogin (2015), whereas the “early childhood phase” spans from 3 to 12 years.

Due to the fast-changing consumer behavior and basket within each life phase as well as the time-consuming process of monitoring and data collection, the life phases “early childhood” and “adolescence” are considered part of future case studies. Therefore, this paper carries out a Life-LCA case study for the prenatal and infancy phases (0–3 years) to analyze associated environmental impacts in these life cycle phases and develop the Life-LCA method further.

To obtain primary data, the human beings chosen as study objects are a 33-year-old mother living in Germany, her newborn baby, and her 2 ½-year-old infant. This sample was selected based on the children’s age to represent the first 3 years of life in the best way and to reduce uncertainties. The mother lives with her husband and the two children in an apartment in a village near Heilbronn, Germany.

In the following chapters, the materials and methods (see chapter 2) used in this study are presented, followed by the study results (see chapter 3), a discussion and sensitivity analysis (see chapter 4), and finally, a conclusion and outlook (see chapter 5).

## Material and methods

This chapter addresses the goal and scope of the study (see Sect. 2.1), the system boundaries (see Sect. 2.1.1), and the impact assessment method used (see Sect. 2.1.2). Furthermore, the life-cycle inventory is presented in Sect. 2.2, including explanations for data collection, the sample, and allocations (see Sect. 2.2.1). The product cluster system with underlying datasets for calculations is presented in Sect. 2.2.2.

### Goal and scope

This study aims to quantify the environmental impact of an infant (0–3 years), including prenatal burdens, by applying the Life-LCA approach (Goermer et al. 2020).

**Fig. 1:**
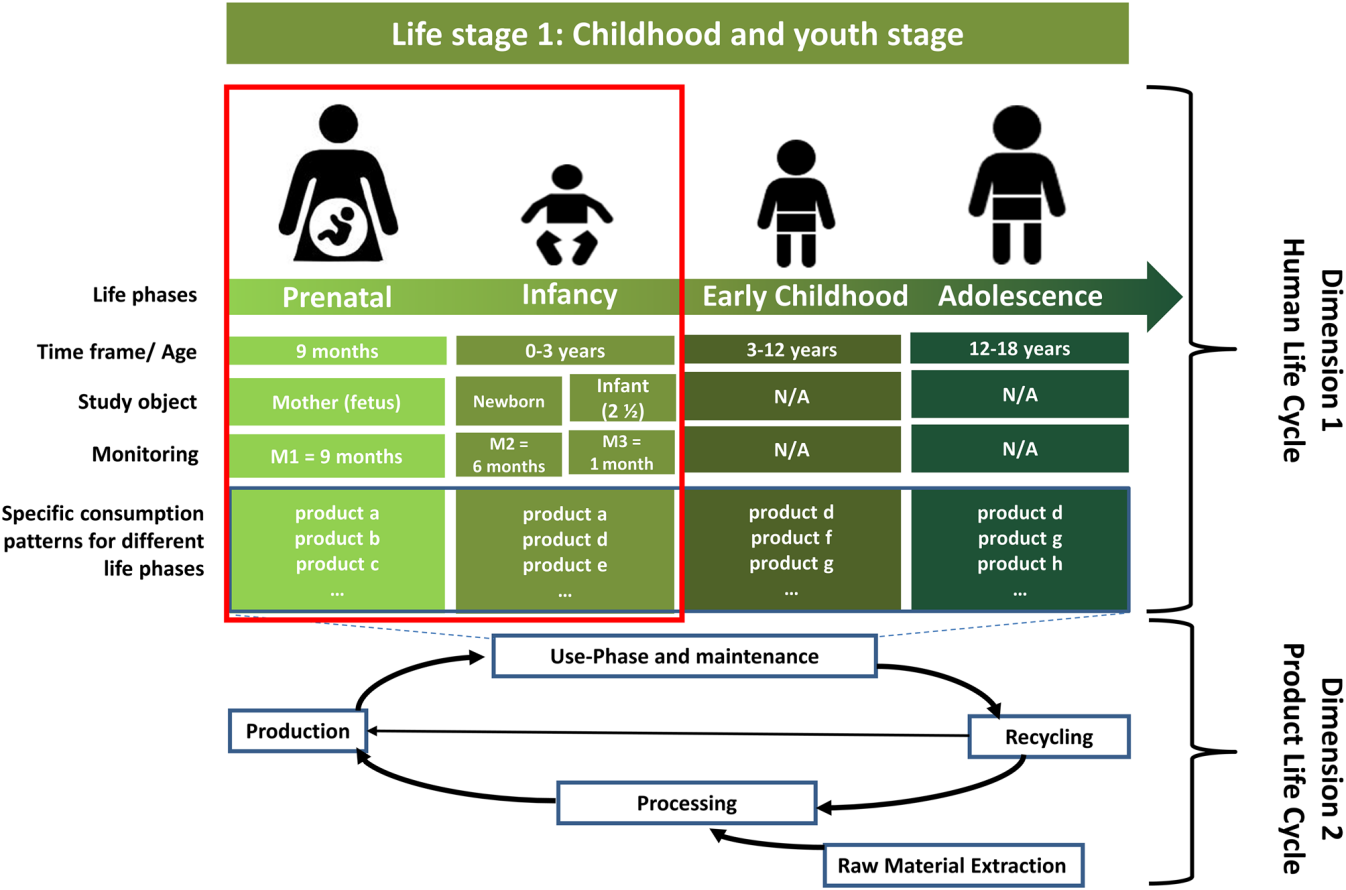
Life stage 1: childhood and youth stage with subdivided life phases and two-dimensional view in Life-LCA, based on Goermer et al. (2020)

The reporting unit is set to an infant, including the prenatal (conception until birth) and infancy phase (0–3 years) of the “childhood and youth stage” (see 2.1.1 and Fig. 1 for a detailed explanation of the subdivision of this life stage). The prenatal phase lasted 262 days from conception to the birth of the infant. The reporting flow describes the range of all consumed products attributable to an infant during both phases. Furthermore, a sensitivity analysis (see Sect. 4.2) is carried out for some products (e.g., single-use diaper vs. reusable diaper) and services (e.g., car vs. public transport) before comparing this case study results with the estimations of the first Life-LCA case study for the “childhood and youth stage” (see Sect. 4.3).

#### System boundaries

For LCAs of human beings, the term life cycle perspective can be either referred to as the life of the assessed person itself (“the human life cycle” visualized as dimension 1) or the life of the products the person consumes (“the product life cycle” visualized as dimension 2) (Goermer et al. 2020) (see Fig. 1). A novelty of the Life-LCA methodology presented in this case study is the division of dimension 1 (life stage) into life phases and extending the system boundaries to the prenatal phase, starting emission accounting already with conception. Especially for growing infants and their complex and fast-changing consumer behavior, a subdivision is necessary to assess the time-dependent consumption behavior. Also, in future case studies, such subdivisions should be the standard for other life stages (e.g., old adulthood stage).

This study considers two phases of the first life stage. Therefore, for determining the environmental impacts of the reporting unit, the temporal system boundaries for the higher-level human life cycle (dimension 1) were set to a “prenatal phase” from conception to birth as well as the infancy phase from birth until year three (see Fig. 1). As mentioned earlier, the “early childhood” phase from age three to age 12, as well as the “adolescence phase” (ages 12–18), were excluded from this study. The system boundaries of dimension 2 (the product life cycle) included all products and services used during the prenatal and infancy phase. Thus, the target of the assessment includes all (i) continuously (e.g., food) and (ii) discontinuously (e.g., clothing) consumed products and services as well as (iii) acquis data (e.g., furniture, toys), where primary data collection was feasible. Acquis data refers to consumed products with an average life span of over a year and cannot be covered within a short (weeks, months) monitoring period. All associated environmental impacts of these products and services were covered from cradle-to-grave, i.e., impacts of all products’ life cycle stages (production of raw materials, product production, use, end-of-life).

For end-of-life, the avoided burden (0:100) approach was applied; i.e., credits were given for recycling, while secondary materials carried the burden of primary material production. In addition, Germany’s current recycling rates were used to determine the recycling share of the used materials (Destatis 2021). Further information regarding the study objects and associated monitoring periods for data collection is provided in Sect. 2.2.1.

#### Impact assessment

**Fig. 2:**
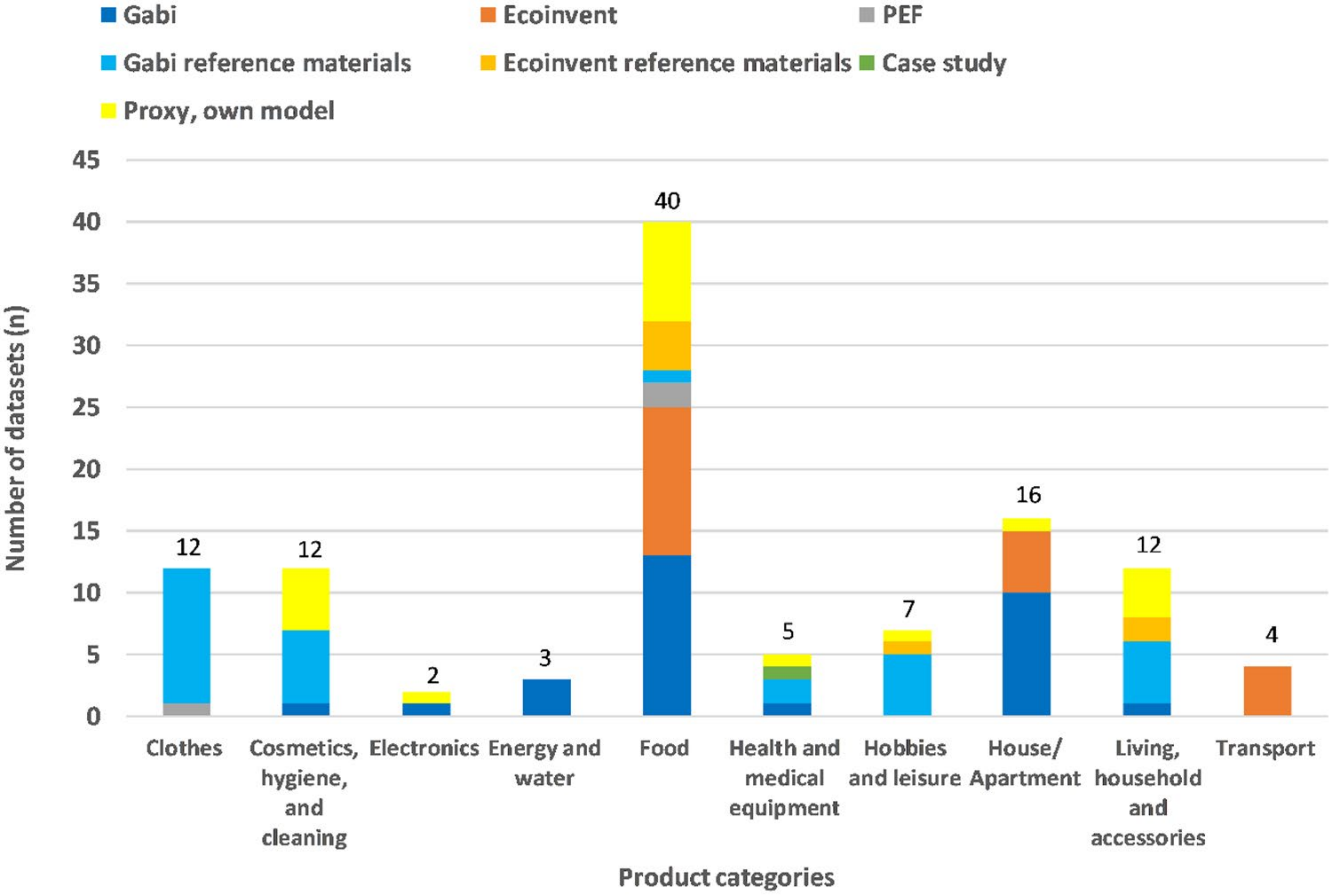
Total number of datasets within the different product categories, including data sources used

The life cycle impact assessment (LCIA) categories used in the case study are climate change (GWP), acidification (AP), eutrophication (EP), and photochemical ozone creation (POCP). The impact categories were selected in line with the first Life-LCA case study. This allows comparing the results of both studies for the “childhood and youth stage” described in Sect. 4.3. Using the same impact categories and assessment methods as for the first Life-LCA case studies enables to conclude the emissions from “cradle to grave” once enough life stages/phases are covered. CML-IA was chosen as it is one of LCA’s most commonly applied approaches for assessing environmental impacts (Bach and Finkbeiner 2017). Additionally, most used “LCA reports and case studies” (see 2.2.2 and Fig. 2: share of 20% regarding all used datasets for modeling), which served as data sources for modeling the missing product categories, used CML-IA. Therefore, the effort of results conversion is reduced (Bossek et al. 2021).

### Life-cycle inventory

This chapter explains the data collection process and describes the sample and several allocations (see Sect. 2.2.1). Then, Sect. 2.2.2 presents the clustering of the different products with their underlying datasets for calculations.

#### Data collection, sample, and allocation

Primary data on consumer behavior in the considered life phases were collected with the help of a sample out of three study objects (see Fig. 1) living in an apartment in a rural area in Germany in the 2020s. The size of the child’s room (12sqm) from the 75 sqm apartment was allocated to the infant, as was the same proportion of the general use of all rooms.

The first monitoring period (M1) included the mother’s pregnancy (2020–06-30 – 2021–03-19). It comprised all data attributable to the fetus. For instance, trips with the car to the gynecologist (product category “transport”) or unique maternity clothes for the mother (product category “clothes”) were included.

In monitoring period two (M2 = 2021–03-19 – 2021–09-19), all data on the consumption of the newborn baby from birth until the age of seven months were collected daily and then extrapolated accordingly to the age of 1 year. The parent’s second infant, around 2 ½ years old, served as an additional study object to cover the second and third years of life. For this purpose, daily consumption in July 2021 was recorded in detail for monitoring period 3 (M3 = 2021–07-01 – 2021–07-31). In addition, acquis data of the older infant (e.g., toys, furniture) were recorded.

The parents documented the consumption on behalf of their two children using the “Life-LCA Excel® data collection sheets” (see TU Berlin Website). The data collection sheets include digital drop-down tables with pre-defined product categories (see Fig. 2) to cover all representative continuously and discontinuously consumed products as well as acquis data. In some cases, the sheets were modified (e.g., the category “hobbies and leisure” was excluded in the prenatal phase). In addition, weekly feedback meetings were held to address questions about data collection.

Some products were weighed using scales as no information was available in literature or online. Sometimes, all products for one cluster got weighed together to save time. For instance, all wooden toys were weighed together and specified as cluster “wooden toys.”

It is assumed that the consumption of both children is the same regardless of gender. Therefore, no gender distinctions are made. Furthermore, the effects of the COVID-19 pandemic in the monitoring year 2021 were not considered. For instance, it was assumed that the infant visited a kindergarten in the third year, as it is usually the case in Germany, even though the kindergarten was partly closed due to the pandemic. Furthermore, all medical examinations from birth until age three were included.

In this study, data on (i) continuously and (ii) discontinuously consumed products of two individuals of the same family were collected separately to gather appropriate data for a successful assessment of the environmental impacts in both considered life phases.

For acquis, however, only the possessions of the older child were recorded in the data collection to avoid double counting (e.g., just the strollers used by the older infant are part of the calculations).

For the considered product categories, the process of data collection, allocation (e.g., the infant’s proportional use of shared products and services (e.g., washing machine) was considered and allocated accordingly) and associated assumptions are described exemplarily in the following to demonstrate the detailed data collection in Life-LCA and enhance understanding of the results in Sects. 3.1 and 3.2. In addition, the complete consumption inventory is available in the supplementary material (SM) (see Table 2. Consumption inventory).

- Cosmetics, hygiene, and cleaning
  Based on M2 and M3, the consumption of all products in this product category was extrapolated to the respective considered years. For instance, diapers were recorded in each monitoring phase and then extrapolated to the years 1–3 accordingly. In addition, the changing size of the diapers with each development step of the child was considered (the weight of the diapers varied from 15 g (diaper size one) to 52 g (diaper size four)). Diapers were changed daily from birth until the eighth month of the third year of life. Onwards until the end of year three, only one diaper (size four) was added per day (this last mentioned diaper was only used for the nighttime).
- Energy

For the prenatal phase, only energy consumption of the devices in the birthing room (e.g., fetal monitor, patient bed, infant warmer system) was considered (Campion et al. (2012)). Thus, the energy consumption per hour of all devices was multiplied by the specific time (5 ½ h) for infant delivery and additional stays in the hospital (modeled in GaBi using the German electricity grid mix).

In the infancy phase, based on the parents’ observations and measurements (weighted measurements to find out the average share of the infant), the infant’s share of energy-intensive machinery (washing machine, dryer, dishwasher) was set as 20%. However, in the first 2 months, the washing machine load and dryer share was 30% due to a higher volume of feces and sputum. The washing machine (approx. 0.95 kWh/60 °C wash cycle) and dryer (approx. 1.46 kWh/dry cycle) were filled and used once daily. The infant’s growth from age two did not result in a higher share of the machine load since feces and spit were reduced by the end of year three.

The dishwasher was included with 0.78 kWh per washing cycle in Eco-Mode (Brand Constructa – Model CP4A00V8). The infant’s share of the dishwasher started in month sixth at 5% (e.g., food storage boxes for baby food). Baby dishes and cutlery were added in the second life year (share 20%). In year three, the infant’s share is assumed to be like an adult’s (33%). The dishwasher was filled once a day on average.

In addition to the energy consumption of the above-mentioned machines, a 20% share of the total electricity consumed in the apartment (based on the electricity bill from 2020) was allocated to the infant to take account of the shared use of all other electronic devices (e.g., baby radiant warmer, the energy consumption of light bulbs) and the electric (floor) heating.

- Food
  For the prenatal phase, a generic food basket was used for extra calories during pregnancy. This basket contains all necessary product clusters for a balanced diet based on statistical data on average food intake from the Robert Koch Institute (Mensink et al. 2007). Alcoholic beverages were excluded. Furthermore, according to the German Nutrition Society (2015), the indicative values for extra energy intake for pregnant women are an additional 250 kcal/day in the second trimester and 500 kcal/day in the third trimester. This accounted for an additional caloric intake of 15% on average during pregnancy based on the mother’s basal metabolic rate.
  For breastfeeding during the first five months of the infancy phase, a plus of 500 kcal/day to the mother’s intake was considered (DGE 2015). At the beginning of the sixth month, the food and water consumption of the infant was included. The sixth month of infant one (M2) was used as a reference to extrapolate food consumption until 1 year. Food consumption in year three was based on M3 when the second infant was 2½ years old.
  Since there was no study object for the second year of life, the parents reported all non-consumed product clusters from the infant for the second year based on their memories. This basket with the remaining product clusters was multiplied by a factor of 0.8 since there was a slightly lower consumption rate than in year three. Drinking water consumption was assumed to be 250 ml a day from month six to age three, based on Mensink et al. (2007). Other beverages (e.g., juice and milk alternatives) were recorded separately.
- Transport
  Trips to the kindergarten and pediatrician were conservatively allocated entirely to the infant. Furthermore, it was assumed that the car rides have no multipurpose (e.g., the infant gets dropped off on the way to the office). One vacation trip with the infant was excluded from the calculations as the parents determined this exclusively. However, such a scenario and possible results are shown in the sensitivity analysis in Sect. 4.2.
- Water
  For infant delivery and additional stays in the hospital, water consumption was assumed to be 400 L per day (including water use for infant delivery, drinking, and washing) (Deutsche Bundesstiftung Umwelt 2020; EnergieAgentur.NRW 2020).
  Furthermore, the baby’s water consumption was determined with a baby bucket (five liters per body wash) for the first six months and then extrapolated to the rest of year one. Furthermore, washing hands and feet twice a day led to water consumption of 0.5 L each time. In the second year, the infant was bathed once a week in a small bathtub (capacity of 100 L). Furthermore, two showers (72 L per six min. shower) were added per week (UGR 2020). From the third year onwards, the infant was bathed only once a month but showered twice per week.
  The washing machine consumed 45 L per washing cycle, whereas the dishwasher only used 13 L. The infant’s share of both machines is the same as for “energy.”

#### Product cluster system and underlying data sources for calculations

Due to the sheer amount of different products used, not all of them could be modeled in detail. Thus, the bottom-up clustering scheme was used for simplification, as described by Goermer et al. (2020), and already successfully applied in the first Life-LCA case study (Bossek et al. 2021). In addition, products for which LCA data are unavailable were assigned to products with similar characteristics for which LCA datasets are available. For instance, different types of cheese (feta, mozzarella) are modeled under dairy products (using the dataset for cheese).

Furthermore, the product categories were only slightly changed in terminology to guarantee better comparison with the first and possible future Life-LCA case studies (e.g., previously, “hobbies, leisure, and pets” was renamed “hobbies and leisure” as the study objects had no pets in this case study). The total number of product categories stayed the same, even though only six out of the ten were calculated for the “prenatal phase.” Over 113 product clusters were established for this case study, from which a quarter was newly modeled (e.g., diapers). These new datasets for the product clusters considering consumer goods in the prenatal and infancy phase expand the overall Life-LCA database. Developing a consistent database with aggregated consumer goods data remains one of the main tasks for improving the Life-LCA method. Therefore, each case study that contributes to this task by providing new modeled datasets to the Life-LCA database, particularly of not yet considered life stages or phases, is a crucial development step to facilitate future calculations. Most of the product clusters were located in the product category “food” (40 products = 35%), as shown in Fig. 2. However, some categories show only a few products because the category contains a limited number of products (e.g., “energy and water” = 3), or the study objects’ consumption was limited to a few products covered by this product category (e.g., baby phone for “electronics”).

The LCA-software GaBi (Sphera 2021), content version “2021.2,” was used to model the individual products and services. As shown in Fig. 2, almost half of the product clusters were modeled with GaBi or Ecoinvent datasets (Ecoinvent 2021) as “reference materials.” The term “reference materials” was used when (a) no specific product datasets were available, (b) no information on the average products’ composition was given, or (c) product-specific case studies were not available. If such a product consisted mainly of one material, it was modeled based on an available average material data set. For instance, a closet with no specific data was modeled using an average dataset for wood mix (in Germany), neglecting smaller parts like screws.

The focus was on (1) common LCA databases such as GaBi (share of 54%) and Ecoinvent (share of 24%), (2) product environmental footprint (PEF) studies (European Commission 2019) (share of 2%), and (3) “LCA reports and case studies” (share of 20%). In contrast to the first Life-LCA case study, the number of datasets based on “case studies” was deliberately reduced to 1%, as existing case studies often do not account for all impact categories and are therefore not an ideal source to fill data gaps (Huber et al. 2022). This led to an increased share of “proxy (own model)” data sets (19%), which are based on a mix of material compositions found on the internet, LCA reports, and case studies. Compared to the first Life-LCA case study, about 35% more data sets were used from common LCA databases. However, GaBi and Ecoinvent databases lack datasets for consumer goods, making the dependency on LCI/LCIA data from LCA reports and case studies still a limiting factor for calculating other impact categories such as “land use” or “water footprint.”

The supplementary material (see section “consumption inventory”) provides a detailed overview of all used datasets per product category and cluster.

## Results

This chapter presents the results of the prenatal (see Sect. 3.1) and infancy (see Sect. 3.2) phases.

### Prenatal phase

**Fig. 3:**
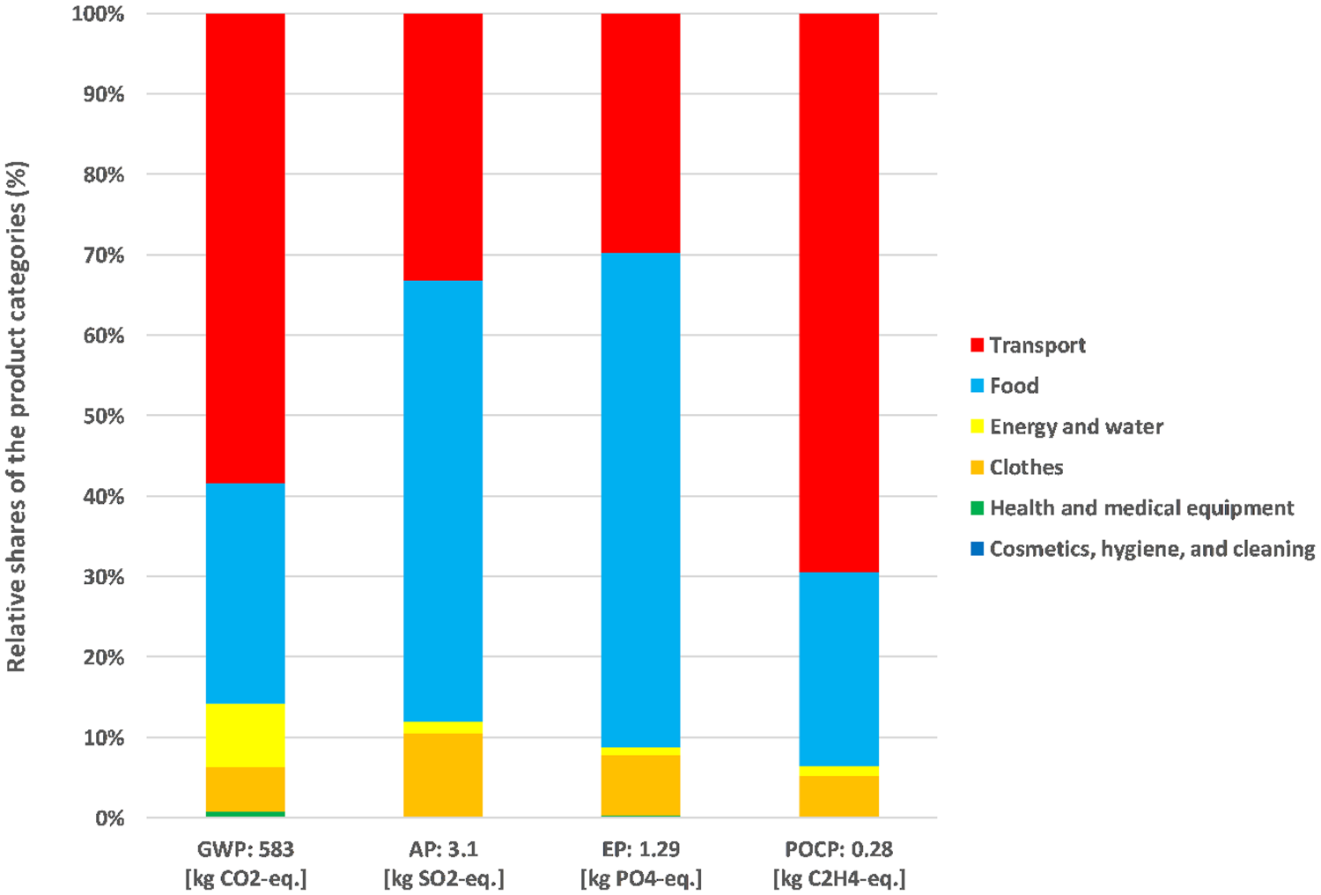
Prenatal phase results and relative shares of product categories

Figure 3 shows the environmental impacts of the infant from conception until giving birth. The results for GWP add up to 583 kg CO_2_-eq, for AP to 3.1 kg SO_2_-eq., for EP to 1.29 kg PO_4_-eq., and for POCP to 0.28 kg C_2_H_4_-eq.

The share of transport is dominant for GWP (58%) and POCP (69%). For “transport,” trips by car to the gynecologist account for the largest share (77% or 700 km from a total of 900 km). These trips include, e.g., all preventive examinations before birth (e.g., ultrasound examinations) or the transport to and from the hospital for infant delivery, including the pick-up of the mother and newborn.

The extra calories during pregnancy for the growth of the fetus, based on a generic food basket (see Sect. 2.2.1), have the second largest share for GWP (27%) and POCP (23%). Although fruits and vegetables have the highest percentage in quantity, dairy products (GWP: 65%) and meat (GWP: 12%) lead to the highest impacts. AP and EP have a total share for “food” of around 60%, also dominated by dairy products and meat. The product categories “transport” and “food” together have a share of around 85% (or higher for AP 88%; EP 92%; and POCP 94%) in the considered impact categories.

The product category “clothes” has a small contribution (5–10%) and is dominated by the five kg of maternity clothes mainly made of cotton. These cotton clothes lead with a contribution of 55% for GWP and 93% for EP.

The impact of the product category “energy and water” is comparatively low for the impact categories AP, EP, and POCP (< 2%). However, for GWP, “energy and water” has an overall share of 8% and is the third most significant contributor in this impact category. The main contributors are infant delivery devices (e.g., fetal monitor, infant warmer system, patient bed) and additional days in the hospital (three days in total). Overall, the time in the hospital (including giving birth and additional stay) caused about 46 kg CO_2_-eq. emissions.

Since only a few products of the product category “cosmetics, hygiene, and cleaning” (pregnancy tests) and “health and medical equipment” (food supplements) were used, their impact (< 1%) across the considered impact categories is low.

### Infancy phase and comparison

The infancy phase accounts for environmental impacts for GWP of 3,428 kg CO_2_-eq, for AP of 19.2 kg SO_2_-eq, for EP of 9.4 kg PO_4_-eq., and for POCP of 1.4 kg C_2_H_4_-eq.

**Fig. 4:**
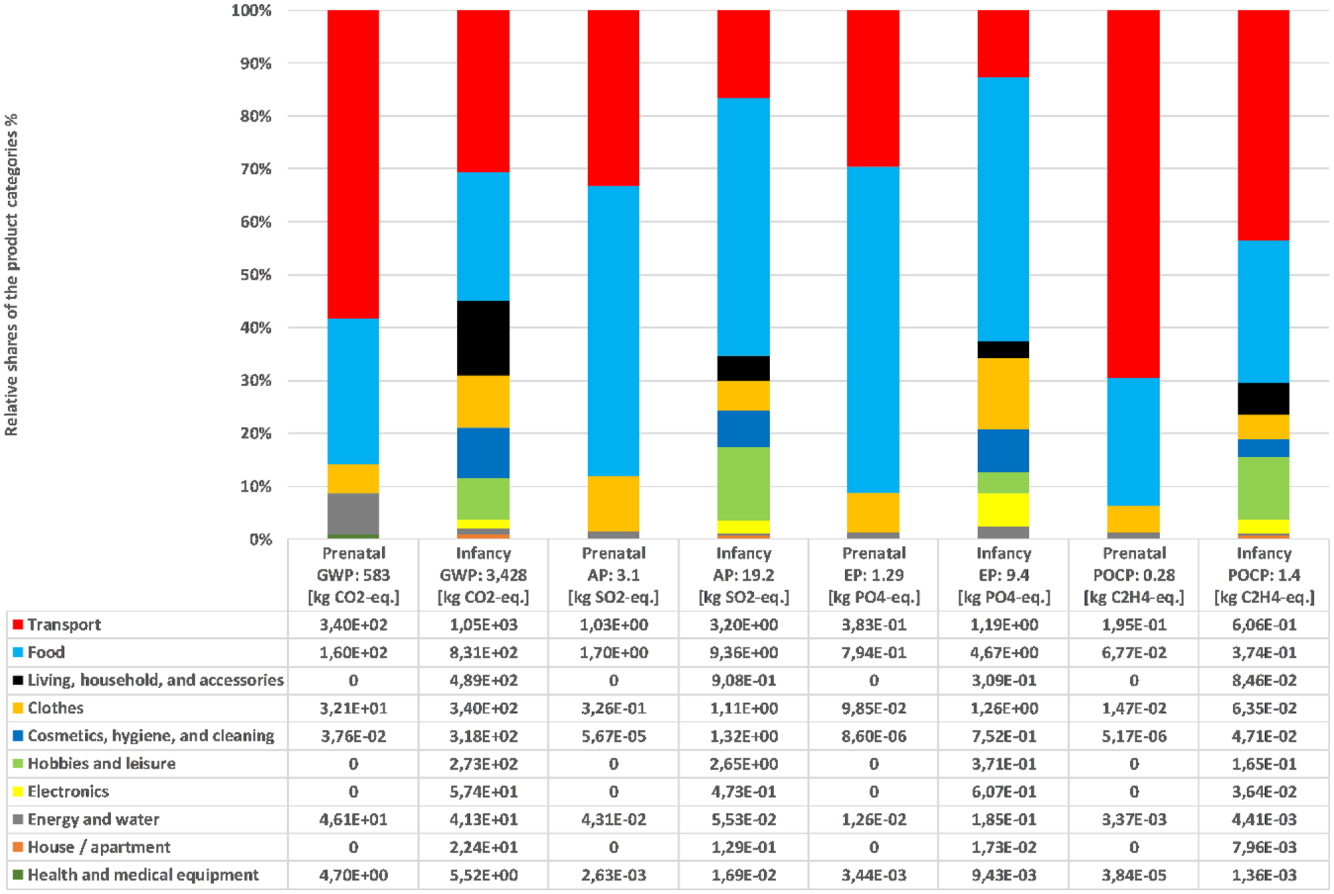
Infancy and prenatal phase results and relative shares of product categories

A direct comparison of the two life phases across all impact categories shows that the four product categories “living, household, and accessories,” “hobbies and leisure,” “electronics,” and “house/apartment” do not have any impact on the prenatal phase (see Fig. 4 – values are “0” for non-suitable product categories). The product categories “transport” and “food” have the highest impacts across all categories in both life phases except for EP (here, “clothes” have a higher impact than “food” in the prenatal phase). In terms of GWP, the prenatal phase has higher shares for “health and medical equipment” and “energy and water,” in contrast to the infancy phase. In total, both life phases together have a GWP of approx. 4t CO_2_-eq., with a share of 15% for the prenatal phase. The same ratio is valid for AP and EP. For POCP, the share of the prenatal phase is slightly higher at 20%. The product category “cosmetics, hygiene, and cleaning” has minor impacts (lowest proportion over all considered product categories) for the prenatal phase; however, for the infancy phase, it holds the fifth out of 10 places due to the high use of diapers.

Infancy phase results per product category

- Transport
  Figure 4 shows that “transport” has the highest share in the impact categories GWP (31%) and POCP (44%). The regular trips by car (12 km, four times a week) to the kindergarten account for the highest share for GWP and POCP (around 80%). All medical examinations from birth until age three have a share of around 7% for GWP and POCP.

- Food
  The category “food” dominates AP and EP with a 50% share and is the second dominant product category for GWP (24%). On average, 250 g of food products per day were consumed after the sixth month. Meat (including beef, poultry, and pork) has the highest impact (45%) on GWP, followed by dairy products, with a share of 35%. Nuts and bakery products follow in third place with 5% each. For EP, nuts and bakery products are more dominant, with a share of 15% and 8%, respectively. The results for all product clusters in each impact category can be seen in detail in the supplementary material (see 1. LCIA results).
- Living, household, and accessories
  The product category “living, household, and accessories” has a total share of 14% for GWP, whereas for AP, EP, and POCP, the share is around 3–5%. The product cluster “furniture, wood” (e.g., shelves, table, bed, wardrobe with a total quantity of 300 kg) show the highest share across all impact categories (on average 35%). The second-largest share (26%) has the product cluster “Baby, accessories PET,” dominated by the infant carrier seats. Third, are the product clusters “bedroom utensils, cotton” (containing carpets, bedsheets, and pillows) and a modeled “baby buggy,” which both contribute 8%.
- Hobbies and leisure
  The product category “hobbies and leisure” has the third-largest share after “transport” and “food” for the impact categories AP (14%) and POCP (12%), as shown in Fig. 4. The main contributors are the infant’s bicycles (two running bikes in different sizes and one tricycle), which account for 75% of the environmental impact for GWP and 50% for AP and POCP. In contrast, toys made of cotton dominate “hobbies and leisure” for EP with a share of 50%, followed by books with 33% (see SM).
- Clothes
  The product category “clothes” shows the third-highest share for the impact category EP with 14%, 10% for GWP, and around 6% for AP and EP. The product category is dominated by the large number (quantity: 64%) of clothes made of cotton (share of “baby clothes, cotton” in the impact category POCP 60% and EP 94%). Clothing made of polyamide accounts for a GWP of approx. 15%. Linen and leather (pair of shoes) clothes were only used in small amounts and therefore did not contribute significantly to the overall results for “clothes” (share of 0–1%).
- Cosmetics, hygiene, and cleaning
  Whereas impacts of “cosmetics, hygiene, and cleaning” were low (share < 1%) in the first Life-LCA case study, in this case study, they have a share of 10% for GWP in the infancy phase due to the high amount of diapers. In three years, 156 kg of single-use diapers (approx. 4,500 diapers) were used, contributing 70% to GWP. Besides diapers, “care accessories, cotton” (mostly nursing pads, towels, and wipes) have a 13% share of GWP. The third biggest share is “cleaning, paper” for POCP (14%) and GWP (around 5%).
- Electronics
  For “electronics,” the relative share is 7% for EP, followed by AP and POCP (both 3%) and then GWP (2%). A diaper-changing table heater has a share for “electronics” of around 65% over all impact categories. Followed by a bottle warmer and baby monitor with impacts from 15 and 20% for GWP. The impact of their use phase is covered under “energy and water.”
- Energy and water
  The product category “energy and water” has a share of 2% for GWP and EP. POCP and AP have a share of 0.3%. The family derives electricity from a certified renewable energy provider (hydropower). Nearly half of the infant’s energy consumption is composed of the proportional use of the dryer (25%), washing machine (9%), and dishwasher (6%). The infant’s share of the family’s total energy consumption is 32% on average for the first three years.
  In total, the infant used about 36,500 L of water (excluding drinking water) for showering (36%) using the washing machine (28%), bathtub (17%), and dishwasher (8%).
  The relative share of the remaining product categories amounts to 0–1%. For “house/apartment,” the cluster “wall stones” show the major contribution with 45%. Medical oil (60%) and the purchase of a breast pump (27%) dominate the product category “health and medical equipment.”

## Discussion

In this chapter, the results and procedure of the case study are examined in Sect. 4.1. Furthermore, a sensitivity analysis is presented in Sect. 4.2. Section 4.3 presents a comparison of the infancy phase results with the first Life-LCA case study.

### General discussion of the results and procedure

The results of the two life phases have shown that consumer behavior changes significantly per life phase concerning the number of different products (increase in the consumer basket) and quantity consumed. It is suggested that in future studies, each life stage is subdivided into life phases to consider these changing consumption behaviors under socio-psychological models of human development or consumer studies. In addition, using data from different study objects (in this case study: a newborn baby and 2 ½-year-old infant) with the same attributes (e.g., same family, social class, country) for calculating one life phase could further decrease uncertainties.

Collecting primary data, especially during the “childhood and youth stage,” was essential to make a valid statement about consumer behavior in this life stage per product category and enhance data quality by further expanding the database. In some cases, primary data collection was not feasible. For instance, medical masks, gloves, or disinfectants were not considered during childbirth because data collection at that moment but also retroactive was not possible. Furthermore, for the prenatal phase, any pre-birth examinations with devices (e.g., ultrasound) were omitted as data on machine types, and their energy consumption was unavailable. Also, primary data collection revealed that more diapers were used than on average (Diaperplanner 2021), highlighting its importance compared to general assumptions.

Furthermore, the chosen study objects were deliberately not representative of Germany, as the Life-LCA method determined the impacts on individuals. The initial parameters would have to be similar for a generalization to obtain comparable results for the considered product categories. For instance, whether the infant is growing up in an urban or rural area would lead to different transport emissions (e.g., more accessible public transport in cities). A strong influence is also the income class of the parents, which affects the size of the living space and overall consumption patterns (German Environment Agency 2016). Carrying out a Life-LCA for individuals living in a different country or culture also influences the results. For instance, electronic products such as a “baby phone” or “baby radiant warmer” would be redundant or considered luxury products in warmer or poorer countries than Germany. Further influencing factors are the electricity mix used and the housing situation (e.g., old vs. new building (good insulation) or house vs. apartment).

Even though the consumption of “food” by the study objects of this case study is similar to an average infant, a change in the diet itself, e.g., a more vegetarian and seasonal one, could influence the results. These examples show that general data cannot reflect each study object’s unique consumption behavior. In addition, using alternative products (e.g., reusable diapers) could also affect the results significantly. Some of the above alternatives are examined in a sensitivity analysis (see Sect. 4.2).

Future studies with different social or cultural backgrounds and consumer behaviors and lifestyles are suggested to develop the Life-LCA method further and gain new insights.

The methodology (see Sect. 2.2.1) to account for the mother’s food intake during pregnancy and breastfeeding contains some uncertainties as secondary data sources were used. It should be analyzed if secondary data (also for other product categories) in other life stages and phases in future studies could be an option if the individual aspects are not the study's focus (individual Life-LCA vs. Lifestyle-LCA (Goermer et al. 2020)) or primary data collection is not feasible. Furthermore, the time for primary data collection could be reduced a lot as data collection on food is the most time-consuming effort for the study objects considering all product categories. However, a methodology to determine data quality would be necessary to classify specific sources of secondary data. In addition, the quality of the collected data and associated results depend on the accuracy and motivation of the study objects. Thus, in terms of the system boundaries of the study, the study object(s) should be selected based on available time.

Furthermore, this Life-LCA case study applied a prospective approach from conception onwards based on primary data, which is essential to classify the results with reduced uncertainty. It has to be mentioned that the time between the mother's conception and her realization that she was pregnant was considered in the calculations.

The criteria to determine cut-offs for product categories (based on certain hotspots) or life phases should be developed depending on the system boundaries of future Life-LCA studies. For instance, this study has shown that prenatal burdens are essential when setting the system boundaries to the first two life phases or the “childhood and youth stage.”

Although the study objects were examined for 15 months (nine months of pregnancy and six months after birth), there are uncertainties in the extrapolations and downward calculations for some product categories (e.g., “food”). In addition, the gender of the study objects was not considered in this study but might play an increasing role as the infant develops (e.g., food intake, clothes) and should be considered for other life phases (e.g., adolescence) and stages (e.g., early adulthood stage).

Furthermore, the results showed that AP and EP are more sensitive to the product category “food” (especially agricultural products). Influencing factors on the sensitivity are the type of consumed “product,” its particular impacts, as well as its consumed quantity. POCP is sensitive to the product category “transport” and its associated products (e.g., car) (see Fig. 4). In the supplementary material, which factors influence the different impact categories and to which extent is presented in detail.

The life phase with the highest sensitivity regarding its environmental impacts cannot be identified because results highly depend on individual consumer choices.

This study used a relatively conservative approach in which all environmental impacts were allocated entirely to the infant. However, the question remains on how to allocate the impacts adequately between the infant and parents in a full Life-LCA from “conception to grave.” Additionally, in the context of a family, considering siblings and other generations, the question of allocation is relevant. Chapter 2.2.1 presents several assumptions and allocation options applied in this study. However, for Life-LCA, a methodology for an adequate allocation of burdens between family members considering all life stages (from childhood to old adulthood stage) and the degree of free decision (e.g., how to allocate burdens in case of sickness) is missing. Establishing such Life-LCA allocation rules per life stage/phase and product categories as well as external parameters (e.g., how is the car ride allocated if parents take an infant to the kindergarten on their way to the office) should be part of future methodological development.

Moreover, one aim of this study is to promote environmental awareness among readers, especially young families. The decision to have children is associated with unavoidable future emissions, which continue over generations and can contribute to an individual’s carbon legacy depending on the allocation of the associated impacts (Murtaugh and Schlax 2009). Given the additional emissions involved when giving birth to a child, the question of whether to have children quickly drifts into ethical discussions. One argument, of course, is that procreation is a fundamental meaning of life. Thus, this highlights the importance of this study as it helps young families reduce emissions after ovulation through sustainable consumer choices by identifying hotspots and showing alternatives, such as in the following sensitivity analysis.

### Sensitivity analysis

In the following, a sensitivity analysis is carried out by modeling product alternatives in different product categories:

- “Clothes,” “hobbies and leisure,”; and “living, household, and accessories”
  Where possible, secondary materials are modeled instead of primary materials, e.g., “cotton fibers from recycled clothes” instead of “standard cotton fibers” or “recycled wood” instead of primary material for furniture. These changes lead to a reduction of approximately − 75% for GWP for “living, household, and accessories” and “clothes” and a lower reduction of − 10% for “hobbies and leisure.” Moreover, the use of recycled clothes leads to a -92% reduction in EP.
- Cosmetics, hygiene, and cleaning

One alternative to common diapers are reusable diapers made from bamboo fibers (Shanmugasundaram and Gowda 2011). Thus, a bamboo diaper without thermoplastic polyurethane adhesive (Mendoza et al. 2019) is modeled as an alternative diaper for the product category “cosmetics, hygiene, and accessories.” This new product reduces the number of diapers to be worn to 12 (total weight reduction of 99.6%). Therefore, this product cluster is not prominent anymore for “cosmetics, hygiene, and cleaning.” However, “energy and water” impacts double in all impact categories, as the reusable diapers must be washed and dried daily. In this case study, this does not significantly affect the results as the family receives 100% renewable electricity. If, however, Germany’s electricity grid mix is modeled, the results amount to 3,560 kg CO_2_-eq. (diapers share: 65% (2,320 kg CO_2_-eq.)). Thus, the diapers show results equivalent to “transport” and “food.”

- Energy
  The family’s energy consumption was modeled as 100% hydropower. However, due to the challenges of double-counting renewable energy (Holzapfel et al. 2022), other electricity mixes might be used for modeling. For example, if the “German electricity grid mix” is used, an increase in the environmental impacts of the category “energy and water” will be noticed, which would make this category the most significant contributor to GWP (currently seventh-largest: 1.2%) with a share of 27% in the infancy phase.
- Food
  A vegan diet could reduce all impacts by up to 65–79%. For this alternative, the consumption quantity is not changed in the modeling, but, for example, the egg (dough) and pork filling of the “Maultasche” (= special German pasta with filling) is substituted with a vegan alternative. Furthermore, other non-vegan product clusters are substituted with vegan clusters (e.g., “meal vegetarian” is substituted with “meal vegan”). It is assumed that the reduced amount of meat and other dairy products (42 kg) leads to three times the consumption of other products in the product category “food” (mainly fruits and vegetables), as observed in the first Life-LCA case study. This increased amount is evenly distributed among the remaining 23 food clusters.
  Furthermore, formula feeding instead of breastfeeding is modeled. Among other production steps, formula milk requires farming, storage, pasteurization, drying, cooling, packaging, and shipping (Linnecar et al. 2014). An assumed average consumption of formula milk of + 500 kcal per day (see Sect. 2.2.1) for the observed five-month breastfeeding period in this study would result in 75,000 kcal, equal to 13 kg of milk powder and 100 L of water for mixing. For GWP, this results in 366 kg CO_2_-eq, comparable to the emissions of the complete product category “transport” for the prenatal phase based on 700 km (gasoline car).
- Transport
  Savings for GWP and POCP of -70% are possible if the study objects travel all distances by bus instead of a gasoline car (transport to and from the hospital is considered with a taxi in this scenario).
  The results in Sect. 3.2 did not include a vacation trip in the third year of life, as this is determined solely by the parents. If this trip’s environmental burden were considered and shared equally between parents and the infant, emissions for “transport” would increase by 40%. The share of transport for GWP regarding all product categories is then 10% higher for the infancy phase.

### Comparison of the infancy phase results with the approximation of the first Life-LCA case study

In the first Life-LCA case study, consumption in the “childhood and youth stage” was determined based on the assumption that every year of the infancy phase (from birth to 17 years) contributes 50% of the calculated baseline year (48th year) (estimated 4,500 kg CO_2_-eq. per year). Transport emissions of the study object were excluded in the first Life-LCA case study because the study object was driven instead of being the driver. However, this study has shown that transport emissions lead to impacts of approx. 30% of the overall GWP in the infancy phase and therefore should be considered in future studies. For consistent comparison, transport emissions of the infancy phase are not considered. On a 1-year basis, the infancy phase leads to average emissions of about 800 kg CO_2_-eq. The estimated 4,500 kg CO_2_-eq. per year from the first Life-LCA case study seems overestimated by roughly six times the impact (when including the transport emissions (1,150 kg CO_2_-eq.), impacts are still overestimated by roughly four times). However, there is a correlation when comparing the emissions based on the weight of the study object instead of time (on a yearly basis, including the weight of the infant at three years of age (15 kg) compared with an adult (75 kg)). Thus, the case study has shown that primary data collection and detailed consideration of individual life phases are essential to validate generic assumptions. However, the identified connection should be further investigated and validated in future case studies, especially for the pending life phases in the “childhood and youth stage.”

## Conclusion

The presented Life-LCA case study of an infant used a prospective (conception to 3 years) instead of a retrospective approach (49th year to birth) as done in the first Life-LCA case study. Using this approach and developing a new set of specific datasets for a shorter period of three years while collecting data on several study objects for the infancy phase reduced the uncertainties of the results compared to the first Life-LCA case study. Focusing on new life phases has led to a subdivision of the “childhood and youth stage” and an extension of the system boundaries. The case study results show the importance of primary data collection in an individual Life-LCA for evaluating generic assumptions. Furthermore, the sensitivity analysis revealed possible reduction potentials through product alternatives, which are helpful for future sustainable consumer choices. New datasets for particular consumer goods in both life phases expanded the Life-LCA database and added value, data quality, and the base for future studies in the “childhood and youth stage.” Nevertheless, developing a consistently available database with aggregated consumer goods data remains one of the main tasks for improving the Life-LCA method.

Furthermore, future studies should include a methodology for allocating impacts between study objects (e.g., family members) and external parameters (work-life vs. private life) under specific rules and data quality requirements for Life-LCA. In addition, further research on childhood (ages 3–12) and adolescence (ages 12–18) is necessary to derive reliable statements about the environmental impacts of the whole “childhood and youth stage.”

## Supplementary Information

Below is the link to the electronic supplementary material.

Supplementary file1 (XLSX 174 KB)

## Data Availability

All data generated and analyzed during this study are included in the published article and its supplementary information files.
